# Prediction of Potential Small Molecule-Associated MicroRNAs Using Graphlet Interaction

**DOI:** 10.3389/fphar.2018.01152

**Published:** 2018-10-15

**Authors:** Na-Na Guan, Ya-Zhou Sun, Zhong Ming, Jian-Qiang Li, Xing Chen

**Affiliations:** ^1^College of Computer Science and Software Engineering, Shenzhen University, Shenzhen, China; ^2^National Engineering Laboratory for Big Data System Computing Technology, Shenzhen University, Shenzhen, China; ^3^School of Information and Control Engineering, China University of Mining and Technology, Xuzhou, China

**Keywords:** small molecule, microRNA, association prediction, graphlet interaction, similarity calculation

## Abstract

MicroRNAs (miRNAs) have been proved to be targeted by the small molecules recently, which made using small molecules to target miRNAs become a possible therapy for human diseases. Therefore, it is very meaningful to investigate the relationships between small molecules and miRNAs, which is still yet in the newly-developing stage. In this paper, we presented a prediction model of Graphlet Interaction based inference for Small Molecule-MiRNA Association prediction (GISMMA) by combining small molecule similarity network, miRNA similarity network and known small molecule-miRNA association network. This model described the complex relationship between two small molecules or between two miRNAs using graphlet interaction which consists of 28 isomers. The association score between a small molecule and a miRNA was calculated based on counting the numbers of graphlet interaction throughout the small molecule similarity network and the miRNA similarity network, respectively. Global and two types of local leave-one-out cross validation (LOOCV) as well as five-fold cross validation were implemented in two datasets to evaluate GISMMA. For Dataset 1, the AUCs are 0.9291 for global LOOCV, 0.9505, and 0.7702 for two local LOOCVs, 0.9263 ± 0.0026 for five-fold cross validation; for Dataset 2, the AUCs are 0.8203, 0.8640, 0.6591, and 0.8554 ± 0.0063, in turn. In case study for small molecules, 5-Fluorouracil, 17β-Estradiol and 5-Aza-2′-deoxycytidine, the numbers of top 50 miRNAs predicted by GISMMA and validated to be related to these three small molecules by experimental literatures are in turn 30, 29, and 25. Based on the results from cross validations and case studies, it is easy to realize the excellent performance of GISMMA.

## Introduction

MicroRNAs (miRNAs) are a family of small non-coding RNAs, having about 22 nucleotides in length, which regulate gene expression at a post-transcriptional level ( Ambros, 2003). The first miRNA was discovered over 30 years ago in the *Caenorhabditis elegans*. Subsequently, thousands of miRNAs have been discovered in many organisms, and there are currently 2588 annotated miRNAs in the human genome ( Kozomara and Griffiths-Jones, 2014). MiRNAs can simultaneously regulate the expression of hundreds of genes due to the fact that their nucleotide pairing by complementarity is imperfect ( He and Hannon, 2004). In this manner, they play a critical role in a variety of crucial processes such as tissue development, morphogenesis, apoptosis, signal transduction pathways, etc., ( Esquela-Kerscher and Slack, 2006; Spizzo et al., 2009; Wang and Lee, 2009). This additionally implicates them in an array of disease associated processes. The development of large-expression screens has been proven useful in identifying novel miRNAs involved in diseases, which could potentially become an attractive therapeutic target ( Monroig and Calin, 2013; Chen et al., 2017a, 2018a, b, c; Matsui and Corey, 2017).

Regulation of miRNAs by small molecules is an efficient mean to modulate endogenous miRNA function and to treat miRNA-related diseases ( Xia et al., 2015). Small molecules have been thoroughly used with clinical applications for numerous diseases ( Zhang et al., 2009). However, drug discovery and development are currently an extremely long process, which takes approximately 10–15 years ( Monroig Pdel et al., 2015). Also, drug production results in an incredible economic burden and patients end up having to pay exaggerated prices for their treatments ( Chen et al., 2015; Monroig Pdel et al., 2015). The use of chemical compounds that are already FDA approved to treat a specific disease would accelerate the process of completing toxicological studies and clinical trials in order to apply them to other diseases. It would shorten both money expenses and time consuming processes.

As miRNAs have been associated with many diseases ( Chen et al., 2017b), the development of small-molecule drugs targeting specific miRNAs seems to be a promising approach to meet the challenge ( Monroig Pdel et al., 2015). Small molecule may modulate the expression of miRNAs by either activating or repressing their transcription ( Xia et al., 2015). Transcriptional inhibitors were identified by completing a small molecule screen in which a 3′ UTR complementary to miR-21 was inserted into a luciferase mRNA reporter ( Gumireddy et al., 2008). This study identified a type of diazobenzene as miR-21 transcriptional inhibitors ( Gumireddy et al., 2008). Small molecules were also discovered to modulate transcription of miR-122, a highly expressed and liver-specific miRNA whose aberrant expression is associated with hepatocellular carcinoma ( Thomas and Deiters, 2013). Two small molecules that inhibit transcription and another small molecule that promotes transcription of pri-miR-122 were identified using a luciferase reporter system ( Thomas and Deiters, 2013). The examples above show that miRNA expression can be altered with small molecules, providing promise to expand miRNAs from diagnostic signatures of disease to therapeutic targets. Therefore, the prediction of associations between small molecules and miRNAs could promote the drug repurposing for miRNA-related diseases. Besides, since the regulation of miRNA expression can be caused by targeting miRNAs directly ( Zhang et al., 2010) or by targeting the relative proteins ( Lim et al., 2016), identifying the small molecule-miRNA associations would be conductive to the drug discovery. However, experimental methods to study the small molecular-miRNA association are expensive and time-consuming, which makes it urgent to develop computational approaches to provide reliable predictions that can give some guidance to experiments.

Recently, several computational models have been proposed to investigate the relations between small molecules and miRNAs. For example, Jiang et al. (2012) proposed a high-throughput method to investigate the biological connections between small molecules and miRNAs in 23 human cancers based on transcriptional responses, which was the first model to systematically study the associations between bioactive small molecules and miRNAs. They constructed a complex Small molecule and MiRNA Network (SMirN) for each cancer and explored the molecular and functional features for small molecule modules, as well as miRNA modules for each cancer type. Each module of small molecular was linked to a miRNA, and each module of miRNA was connected with one small molecular. One of the advantages of this method is that it does not need to know the information of small molecule structure or miRNA structure in advance. However, the reliability of the approach was limited due to the small data of transcriptional response to genome-wide miRNA perturbations. Furthermore, Meng et al. (2014) built a bioactive Small molecule and miRNA association Network in Alzheimer’s Disease (SmiRN-AD) through comparing the gene expression profiles after bioactive small molecule treating with the AD-related miRNA (ADM) regulating expressions, to get the scores of associations between small molecules and ADMs. Besides, the positive and negative associations were identified to investigate the biological insights of the SimRN-AD. Recently, Wang et al. (2016) developed another method to identify small molecule-miRNA associations based on their functional similarity. They searched the functional link of each small molecule-miRNA pair by calculating Gene Ontology enrichment after identifying differentially expressed genes for small molecules and miRNAs. Compared with previous models based on transcriptional responses, this method is more repeatable by using functional associations. Additionally, Lv et al. (2015) presented a novel computational model to predict potential associations between small molecules and miRNAs. They implemented the random walk with restart algorithm on an comprehensive network, which was established by combining small molecule similarity, miRNA similarity, as well as known small molecule-miRNA associations. Especially, this model can predict the novel related miRNAs for small molecules without any known associated miRNAs. However, it has too many adjustable parameters that need to be affirmed. Moreover, Li et al. (2016) developed a network based framework called predictive Small Molecule-miRNA Network-Based Inference (SMiR-NBI), to investigate the underlying regulations of anticancer drugs on miRNAs. This model constructed a heterogeneous network that was composed of drugs, miRNAs and genes to conduct a network based algorithm. It is mentionable that the accuracy of this method is quite high even it only depended on the network topology information. However, SMiR-NBI could not be applied to prediction of isolated miRNAs that have no interlinked small molecules. Besides, it failed to predict potential miRNAs associated with small molecules that had different dose-responses, due to lack of known data.

So far, the number of computational models is still not satisfying for the prediction of novel associations between small molecules and miRNAs. Moreover, there are still some limitations existing in the previous models. In order to predict potential small molecule-miRNA associations more effectively and reliably, in this paper, we presented the Graphlet Interaction based inference for Small Molecule-MiRNA Association prediction (GISMMA). In this model, the similarity of small molecules and the similarity of miRNAs were combined with known associations between small molecules and miRNAs in two different datasets, which were labeled with Dataset 1 and Dataset 2. In Dataset 1, only a fraction of small molecules and miRNAs were involved in known small molecule-miRNA associations, whereas in Dataset 2 all small molecules and miRNAs were implicated in known small molecule-miRNA associations. Based on the measuring of graphlet interaction between any two nodes on the network of small molecules and on the network of miRNAs, respectively, we can compute the correlation scores of small molecule-miRNA pairs. We have implemented leave-one-out cross validation (LOOCV) and five-fold cross validation to evaluate the performance of GISMMA. The AUCs of global LOOCV are 0.9291 and 0.8203 for Dataset 1 and Dataset 2, respectively; the AUCs of local LOOCV by ranking the small molecules for each fixed miRNA are, respectively 0.9505 and 0.8640 for the two datasets; the AUCs of local LOOCV by ranking the miRNAs for each fixed small molecule are, respectively 0.7702 and 0.6591 for the two datasets. And the average AUCs and standard deviations of five-fold cross validations are 0.9263 ± 0.0026 and 0.8088 ± 0.0044 for the two datasets, respectively. In case study, small molecule was set as a new one by turning all known related miRNAs into unknown ones. GISMMA was then applied to predicting latent related miRNAs for each small molecule based on the Dataset 1. For the small molecules, 5-Fluorouracil, 17β-Estradiol and 5-Aza-2′-deoxycytidine, there were in turn 30, 29, and 25 out of top 50 predicted miRNAs, which were validated to be associated with these three small molecules by experimental literatures, respectively. The results both in cross validations and case studies have suggested that GISMMA is a powerful and reliable model to predict novel associations between small molecules and miRNAs.

## Materials and Methods

### Small Molecule-miRNA Associations

In this paper, we obtained the known small molecule-miRNA associations from SM2miR (Version 1) ( Liu et al., 2013). The total number of known associations is 664. For comparison of model performance on different datasets, we have constructed two datasets. Dataset 1 consists of 831 small molecules extracted and integrated from SM2miR, DrugBank ( Knox et al., 2011) and PubChem ( Wang et al., 2009), and 541 miRNAs that were collected from SM2miR, HMDD ( Lu et al., 2008), miR2Disease ( Jiang et al., 2009) and PhenomiR ( Jiang et al., 2009; Ruepp et al., 2010). In Dataset 1, there are only 39 small molecules and 286 miRNAs implicated in the 664 known associations, while 792 small molecules and 255 miRNAs are completely new ones without any known associations. Dataset 2 is only composed of those 39 small molecules and 286 miRNAs, which are involved in the known associations. Based on the known data, an adjacency matrix *A* was constructed to represent the relations between small molecules and miRNAs, in which *A*(*i, j*) was set to be 1 if there is an association between small molecule *s*(*i*) and miRNA *m*(*j*), 0 otherwise.

### Small Molecule Similarity

In this paper, according to the method proposed in ( Lv et al., 2015), the small molecule similarity was calculated by integrating four usual small molecule similarities which were side effect based similarity that was computed by Jaccard score using small molecule side effect dataset ( Gottlieb et al., 2011), functional consistency based similarity that was obtained by comparing the function of small molecule target genes ( Lv et al., 2012), chemical structure based similarity that was calculated with the method of chemical structure comparison between any two small molecules ( Hattori et al., 2003), and indication phenotype based similarity that was constructed through identifying phenotype similarity between small molecule related diseases ( Gottlieb et al., 2011). Therefore, the integrated similarity of small molecules can be computed with the following formula:

(1) $$SS=\frac{\beta_{1}S_{S}^{D}+\beta_{2}S_{S}^{T}+\beta_{3}S_{S}^{C}+\beta_{4}S_{S}^{S}}{\sum_{i=1}^{4}\beta_{i}}$$

where, *S^D^_S_, S^T^_S_, S^C^_S_*_,_ and *S^S^_S_* denote the four different similarity types, respectively, i.e., indication phenotype based similarity, functional consistency based similarity, chemical structure based similarity and side effect based similarity, and β_*i*_ (*i* = 1, 2, 3, 4) are the weighs used to balance the different similarity contributions, whose default values were all set as 1.

### MiRNA Similarity

The miRNA similarity we used in this paper was established using the method in ( Lv et al., 2015), by combining functional consistency based similarity that was calculated by comparing the function of miRNA target genes ( Lv et al., 2012) and indication phenotype based similarity that was computed by measuring phenotype similarity between diseases associated with miRNAs ( Gottlieb et al., 2011). Similarly, to reduce the bias of each similarity measurement, the integrated similarity of miRNAs was defined as follows:

(2) $$SM=\frac{\alpha_{1}S_{M}^{D}+\alpha_{2}S_{M}^{T}}{\sum_{j=1}^{2}\alpha_{j}}$$

where, *S^D^_M_* is the indication phenotype based similarity and *S^T^_M_* represents the functional consistency based similarity, and α_*j*_ (*j* = 1, 2) are the weighs of each similarity measurement, which were both set as 1.

### GISMMA

**FIGURE 1:**
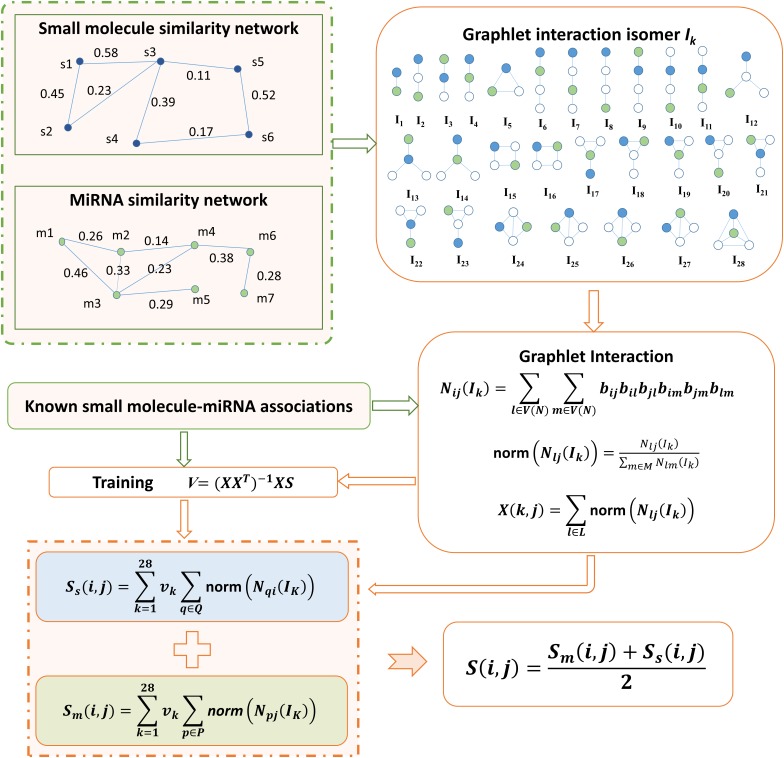
Flowchart of GISMMA model based on graphlet interaction for the prediction of potential small molecule-miRNA associations.

In this study, by integrating small molecule similarity, miRNA similarity and known associations between small molecules and miRNAs, we developed a graphlet interaction based method to predict the potential associations between small molecules and miRNAs, which is motivated by the study of Wang et al. (2014). Prediction code of our model is available at: https://github.com/AnnaGuan/GISMMA/tree/AnnaGuan-patch-1. The concept of graphlet interaction is traced to the definition in ( Wang et al., 2014), which describes the relationship between any two nodes in a graphlet that is a type of subgraph in a large network. As was done in ( Wang et al., 2014), in GISMMA only those graphlets that have 1 to 4 nodes were used, based on which 28 graphlet interaction isomers were constructed, denoted by labels *I*_1_ to *I*_28_ in **Figure 1**. The graphlet interaction isomer depends on the positions of the two involved nodes, which means that the graphlet interaction between two nodes have two different set of isomers. Through counting the number of each isomer, we can represent the graphlet interaction between any two nodes in a network with a vector that contains 28 numbers ( Przulj, 2007; Wang et al., 2014).

We have created a network *NS* to represent the small molecule similarity and a network *NM* to represent the miRNA similarity, where each node in the network denotes a small molecule or a miRNA. The edge with similarity value as its weight exists to link any two nodes that have similarity. The associations between small molecules and miRNAs were investigated in the two similarity networks *NS* and *NM*, respectively.

In the miRNA network *NM*, the number of isomer *I_k_* for graphlet interaction from node *m*(*i*) to node *m*(*j*) can be calculated as follows ( Wang et al., 2014):

(3) $$N_{ij}(I_{k})=\sum_{l\in V(\mathrm{NM})}\sum_{m\in V(NM)}b_{ij}b_{il}b_{jl}b_{im}b_{jm}b_{lm}$$

where *V(NM)* denotes the node set of all nodes in network *NM, l*, and *m* are two nodes different with node *m(i)* and *m(j)*, and *b* is defined as:

(4) $$b_{st}=\{\begin{array}{cc} a_{st} & s\;\mathrm{and}\;t\;\mathrm{has}\;a\;\mathrm{link}\;\mathrm{in}\;I_{k}\\ 1-a_{st} & s\;\mathrm{and}\;t\;\mathrm{has}\;\mathrm{no}\;\mathrm{link}\;\mathrm{in}\;I_{k} \end{array}$$

where, *a_st_* is the edge weight assigned with the similarity value of *m*(*s*) and *m*(*t*). Especially, *a_st_* is 0 when nodes *m*(*s*) and *m*(*t*) have no connection. Then we normalized the graphlet interaction as follows:

(5) $$\mathrm{norm}\;(N_{ij}(I_{k}))=\frac{N_{ij}(I_{k})}{\sum_{m\in M}N_{im}(I_{k})}$$

where *M* contains all other nodes but *m(i)*. Based on the normalized form in equation (5), we can compute the association score of a small molecule-miRNA pair as follows:

(6) $$S_{m}(i,\;j)=\sum_{k=1}^{28}v_{k}\sum_{p\in P(i)}\mathrm{norm}\;(N_{pj}(I_{k}))$$

where *i* denotes a small molecule *s*(*i*) and *j* denotes a miRNA *m*(*j*), *v_k_* is the weight of the *k*th isomer, *P*(*i*) is the set of miRNAs with known associations with small molecule *s*(*i*). By defining the summation of norm in equation (6) as following:

(7) $$X_{m}(k,\;j)=\sum_{p\in P(i)}\mathrm{norm}\;(N_{pj}(I_{k}))$$

we can modify equation (6) into the matrix form as following:

(8) $$S_{m}=X_{m}^{T}V_{m}$$

The weight coefficients *V_m_* can be learnt from known associations by performing a simple linear regression ( Wang et al., 2014), which is given as following:

(9) $$V_{m}={\left(X_{m}X_{m}^{T}\right)}^{-1}X_{m}S_{m}$$

We computed the number of graphlet interaction isomer between two small molecules in the similar way as described in equations (3–5). Then the association score between small molecule *s*(*i*) and miRNA *m*(*j*) can be calculated in the small molecule network *NS* as follows:

(10) $$S_{s}(i,\;j)=\sum_{k=1}^{28}v_{k}\sum_{q\in Q(j)}\mathrm{norm}\;(N_{qi}(I_{k}))$$

where *Q*(*j*) is the set of small molecules that have known associations with miRNA *m*(*j*). Also, the term of summation of norm in equation (10) can be defined with the matrix:

(11) $$X_{s}(k,\;j)=\sum_{q\in Q(j)}\mathrm{norm}\;(N_{qi}(I_{k}))$$

Thus equation (10) was rewritten as:*S_S_* = *X^T^_S_V_S_*, and the undetermined matrix *V_s_* can be obtained by training the model with known association scores:

(12) $$V_{s}={\left(X_{s}X_{s}^{T}\right)}^{-1}X_{s}S_{s}$$

Finally, we calculated the association score between small molecule *s*(*i*) and miRNA *m*(*j*) by combining the scores from *NM* and *NS* in a simple average form as following:

(13) $$S(i,\;j)=\frac{S_{m}(i,\;j)+S_{s}(i,\;j)}{2}$$

## Results

### Performance Evaluation

In this work, two commonly used methods, LOOCV and five-fold cross validation, were implemented to evaluate the performance of GISMMA based on Dataset 1 and Dataset 2, respectively. The LOOCV has three different types including global LOOCV, local LOOCV of ranking small molecules for fixed miRNA and local LOOCV of ranking miRNAs for fixed small molecule. Each confirmed association we collected was taken as the test sample one by one and the rest of known associations were considered as the training samples in LOOCV. Candidate samples in global LOOCV consist of all the small molecule-miRNA pairs that have no known associations. In the case of local, we only consider those small molecules that do not relate to the fixed miRNA or those miRNAs unconnected to the fixed small molecule in the test sample as candidates. The scores as association probabilities were computed using the GISMMA method for both test sample and all candidate samples. Then we ranked them for the corresponding type of LOOCV. The five-fold cross validation was performed in the following steps. Firstly, all the known small molecule-miRNA associations were randomly split into five parts with equal size. Secondly, the five parts take turns to act as the test sample set one after another and the other four parts as the training sample sets; similarly, all small molecule-miRNA pairs that have no known associations play the roles of candidate samples. Thirdly, the test samples as well as the candidate samples were endowed with association scores by GISMMA. Finally, each test sample was picked out in turn to be compared with candidate samples according to their scores. The model was considered to be successfully predict the test sample only when its rank exceeded the given rank threshold.

**FIGURE 2:**
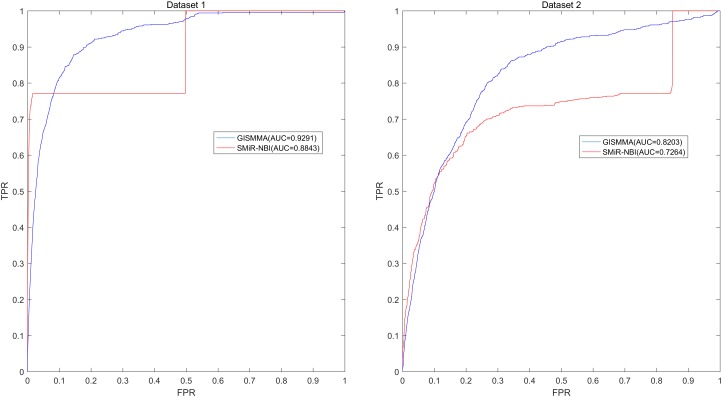
Performance of GISMMA was compared with SMiR-NBI in terms of ROC curve and AUC of global LOOCV for Dataset 1 (left) and Dataset 2 (right). As is shown, GISMMA achieves AUCs of 0.9291 and 0.8203 for Dataset 1 and Dataset 2, respectively, significantly superior to the previous model SMiR-NBI.

**FIGURE 3:**
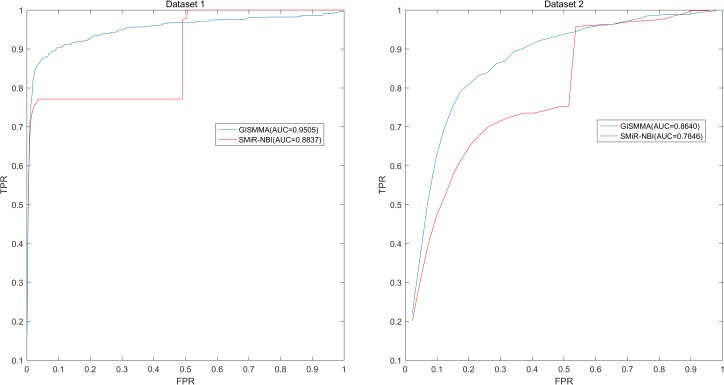
Performance of GISMMA was compared with SMiR-NBI in terms of ROC curve and AUC of local LOOCV of ranking small molecules for fixed miRNA on Dataset 1 (left) and Dataset 2 (right). As is shown, GISMMA achieves AUCs of 0.9505 and 0.8640 for Dataset 1 and Dataset 2, respectively, significantly superior to the previous model SMiR-NBI.

**FIGURE 4:**
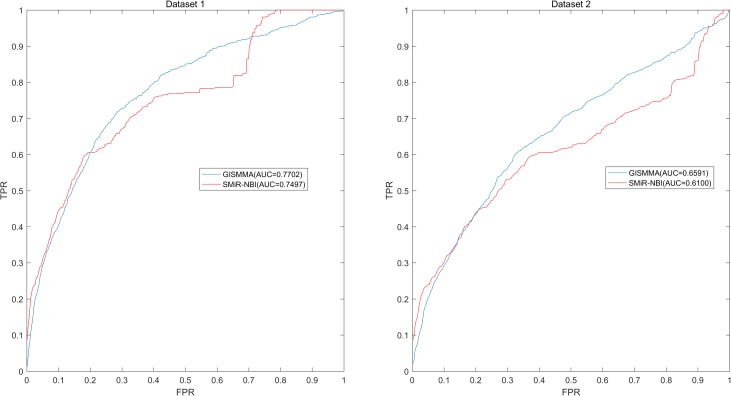
Performance of GISMMA was compared with SMiR-NBI in terms of ROC curve and AUC of local LOOCV of ranking miRNAs for fixed small molecule on Dataset 1 (left) and Dataset 2 (right). As is shown, GISMMA achieves AUCs of 0.7702 and 0.6591 for Dataset 1 and Dataset 2, respectively, significantly superior to the previous model SMiR-NBI.

Based on the ranking, the receiver operating characteristic (ROC) curves were used to illustrate the results of the three types of LOOCV described above, in which the abscissa axis is true positive rate (TPR, sensitivity) and the ordinate axis represents false positive rate (FPR, 1-specificity) for different thresholds given in advance. The sensitivity means the ratio that the positive samples rank above the given threshold, while the specificity is defined as the percentage of candidate samples whose ranks are below the set threshold. The area under the ROC curve (AUC) was correspondingly calculated to estimate the reliability of the GISMMA. When the model correctly predicts all test samples, AUC = 1; but if the model has a random prediction, AUC = 0.5. To make comparison with previous method, we implemented SMiR-NBI ( Li et al., 2016) for global and two types of local LOOCVs, 5-fold cross validation based on the same datasets. The global AUCs of GISMMA for Dataset 1 and Dataset 2 are 0.9291 and 0.8203, respectively, which are shown in **Figure 2** in comparison with previous model SMiR-NBI whose results are 0.8843 and 0.7264, respectively. In the case of local LOOCV of ranking small molecules for fixed miRNA, the AUCs of GISMMA for Dataset 1 and Dataset 2 are 0.9505 and 0.8640, respectively, compared with 0.8837 and 0.7846 of SMiR-NBI, which can be seen in **Figure 3**. The results of local LOOCV of ranking miRNAs for fixed small molecule are shown in **Figure 4**, from which we can see that the AUCs of GISMMA and SMiR-NBI are 0.7702, 0.7497 for Dataset 1, and 0.6591, 0.6100 for Dataset 2, respectively. Besides, in five-fold cross validation, the average AUCs with standard deviations of GISMMA and SMiR-NBI are 0.9263 ± 0.0026, 0.8554 ± 0.0063 for Dataset 1, and 0.8088 ± 0.0044, 0.7104 ± 0.0087 for Dataset 2. The **Table 1** lists the comparison of GISMMA and SMiR-NBI for all AUC results of the four types of cross validations on two datasets. We can make a conclusion from the comparisons that the novel method proposed in this work is more reliable and more effective in predicting potential associations between small molecules and miRNAs.

**Table 1:** The comparison results between GISMMA and SMiR-NBI on AUC values of four cross validations based on two datasets.

DATASET	MODEL	GLOBAL LOOCV	LOCAL LOOCV (fix miRNA)	LOCAL LOOCV (fix SM)	5-FOLD CV
Dataset 1	GISMMA	0.9291	0.9505	0.7702	0.9263 ± 0.0026
	SMiR-NBI	0.8843	0.8837	0.7497	0.8554 ± 0.0063
Dataset 2	GISMMA	0.8203	0.8640	0.6591	0.8088 ± 0.0044
	SMiR-NBI	0.7264	0.7846	0.6100	0.7104 ± 0.0087


### Case Study

Based on the known database and published references in PubMed database, we studied three common small molecules to further evaluate the predictive ability of GISMMA, in which the small molecule in study was set as a new one by taking away its known associations. We ulteriorly observed the number of the experimentally verified miRNAs in the top 50 ones predicted to be related to the three small molecules, respectively.

The small molecular 5-Fluorouracil (5-FU) is a widely used chemotherapeutic drug in colorectal cancer ( Windle et al., 1987). For a long time, the 5-FU-induced cytotoxic effects were thought to result exclusively from its impact on DNA metabolism ( Andreuccetti et al., 1996; Airley, 2009). However, several evidences indicated that the cytotoxic effect of 5-FU also results from its capacity to alter RNA metabolism and mRNA expression ( Longley et al., 2003). Exposure to 5-FU promotes a profound transcriptional reprogramming leading to modification of mRNA and miRNAs expression profiles that contributes in modifying cell fate ( Hernandez-Vargas et al., 2006; Rossi et al., 2007; Shah et al., 2011). After implementing GISMMA, we got the total ranking of potential miRNAs associated with 5-FU. As the result shown, among the top 10 and 50 potential 5-FU-related miRNAs, there were 8 and 30 miRNAs confirmed by experiments, respectively (See **Table 2**). For instance, miR-21 and miR-23a were predicted as the first and fifth candidates for 5-FU, respectively, which were significantly down regulated in comparison between 5-FU treated and control samples in miRNA microarray analysis of 5-FU treated MCF-7 cells ( Shah et al., 2011). Besides, miR-24-1, the third candidate in the ranking list, showed a significantly down regulation in HCT-8 colon cancer cell after exposure to 5-FU ( Zhou et al., 2010). In addition, MiR-27b that ranked the fourth in the prediction list of 5-FU was found to be consistently up regulated in human colon cancer cells HC.21 following exposure to 5-FU *in vitro* ( Rossi et al., 2007).

**Table 2:** Top 50 miRNAs associated with 5-Fluorouracil were predicted by GISMMA based on Dataset 1.

miRNA	Evidence	miRNA	Evidence
hsa-mir-21	26198104	hsa-mir-22	25449431
hsa-mir-324	unconfirmed	hsa-mir-409	unconfirmed
hsa-mir-24-1	26198104	hsa-mir-337	unconfirmed
hsa-mir-27b	26198104	hsa-let-7a-3	26198104
hsa-mir-23a	26198104	hsa-let-7a-2	26198104
hsa-mir-638	26198104	hsa-mir-155	28347920
hsa-mir-27a	26198104	hsa-mir-181b-2	unconfirmed
hsa-let-7b	25789066	hsa-mir-181b-1	unconfirmed
hsa-mir-181a-1	unconfirmed	hsa-mir-15b	26198104
hsa-mir-126	26062749	hsa-let-7i	unconfirmed
hsa-mir-125b-2	unconfirmed	hsa-mir-320a	26198104
hsa-mir-125b-1	unconfirmed	hsa-mir-26a-2	unconfirmed
hsa-mir-124-3	unconfirmed	hsa-mir-328	unconfirmed
hsa-mir-124-2	unconfirmed	hsa-mir-16-2	26198104
hsa-mir-124-1	unconfirmed	hsa-let-7e	26198104
hsa-let-7a-1	26198104	hsa-mir-34b	unconfirmed
hsa-mir-181a-2	24462870	hsa-mir-145	24447928
hsa-mir-24-2	26198104	hsa-mir-200b	26198104
hsa-mir-17	26198104	hsa-let-7c	25951903
hsa-mir-26a-1	unconfirmed	hsa-mir-874	27221209
hsa-mir-16-1	26198104	hsa-mir-650	unconfirmed
hsa-mir-518c	unconfirmed	hsa-mir-501	26198104
hsa-mir-99b	unconfirmed	hsa-mir-500a	unconfirmed
hsa-mir-18a	26198104	hsa-mir-1226	26198104
hsa-mir-663a	26198104	hsa-mir-200c	26198104


*The top 1-25 miRNAs are shown in the first column while the top 26–50 in the second. As a result, 8 and 30 out of top 10 and top 50 were confirmed by the known experimental literatures, respectively.*

The small molecular 17β-Estradiol (E2) is the principal intracellular human estrogen that exerts important effects on the reproductive as well as many other organ systems in both men and women ( Simpson and Santen, 2015). The analogs of estradiol exhibit significant anticancer activity against human breast cancer cell lines ( Sathish Kumar et al., 2014). Estrogens have associations with cancer in target tissues, which is because they have a phenolic ring structure in common with the carcinogenic hydrocarbons ( Ryan, 1982). After implementing GISMMA, we got the total ranking of the E2-associated miRNAs. As the result shown, among the top 10 and 50 potential E2-related miRNAs, there were 5 and 29 miRNAs confirmed by experiments, respectively (See **Table 3**). For example, miR-21, miR-27b, and miR-23a dominated in turn the first, fourth, and fifth places of the ranking list predicted for E2, which were all down regulated after treatment of MCF-7 cells with E2 ( Bhat-Nakshatri et al., 2009; Tilghman et al., 2012). Besides, E2 showed a capacity to down regulate the expression level of miR-21 in breast cancer cells ( Selcuklu et al., 2012).

**Table 3:** Top 50 miRNAs associated with 17β-Estradiol were predicted by GISMMA based on Dataset 1.

miRNA	Evidence	miRNA	Evidence
hsa-mir-21	26198104	hsa-mir-222	24601884
hsa-mir-324	unconfirmed	hsa-mir-31	23143558
hsa-mir-24-1	unconfirmed	hsa-mir-125a	21914226
hsa-mir-27b	26198104	hsa-mir-663a	26198104
hsa-mir-23a	26198104	hsa-mir-22	24715036
hsa-mir-638	26198104	hsa-mir-132	26282993
hsa-mir-27a	26198104	hsa-mir-501	unconfirmed
hsa-mir-181a-1	unconfirmed	hsa-mir-1226	unconfirmed
hsa-mir-24-2	unconfirmed	hsa-mir-328	unconfirmed
hsa-mir-125b-2	unconfirmed	hsa-mir-155	23568502
hsa-mir-125b-1	unconfirmed	hsa-let-7a-3	26198104
hsa-mir-16-1	unconfirmed	hsa-let-7a-2	26198104
hsa-mir-124-3	26198104	hsa-mir-181b-2	unconfirmed
hsa-mir-124-2	26198104	hsa-mir-181b-1	unconfirmed
hsa-mir-124-1	26198104	hsa-mir-26a-2	unconfirmed
hsa-mir-18a	24245576	hsa-mir-15b	26198104
hsa-let-7b	26198104	hsa-mir-20a	21914226
hsa-mir-181a-2	unconfirmed	hsa-mir-29a	22334722
hsa-let-7a-1	26198104	hsa-mir-19a	unconfirmed
hsa-mir-17	26198104	hsa-mir-200b	26198104
hsa-mir-126	26198104	hsa-mir-221	21057537
hsa-mir-26a-1	unconfirmed	hsa-mir-518c	26198104
hsa-mir-320a	27965096	hsa-mir-194-2	unconfirmed
hsa-mir-16-2	unconfirmed	hsa-mir-181d	unconfirmed
hsa-mir-99b	unconfirmed	hsa-mir-197	unconfirmed


*The top 1–25 miRNAs are shown in the first column while the top 26–50 in the second. As a result, 5 and 29 out of top 10 and top 50 were confirmed by the known databases or experimental literatures, respectively.*

The small molecular 5-Aza-2′-deoxycytidine (5-Aza-CdR) is a nucleoside analog inhibitor of DNA methyltransferase (DNMT). It has been used to reverse methylation and reactivate the expression of silenced genes ( Patra and Bettuzzi, 2009). 5-Aza-CdR is able to suppress the growth of various tumors *in vitro*, animal models, and clinical trials including prostate cancer ( Hurtubise and Momparler, 2004; Issa et al., 2004; McCabe et al., 2006). We performed GISMMA on 5-Aza-CdR, and got the total ranking of the predicted miRNAs. As the result shown, among the top 10 and 50 potential 5-Aza-CdR related miRNAs, there were 7 and 25 miRNA-5-Aza-CdR associations confirmed by experiments (See **Table 4**). For example, in the ranking list of miRNAs predicted for 5-Aza-CdR, miR-21, and miR-27b were ranked in the first and fifth position, respectively, both of which showed significant down regulation after 5-Aza-CdR treatment in breast cancer cells ( Radpour et al., 2011). Moreover, miR-24-1 was the fourth miRNA predicted to be associated with 5-Aza-CdR. Microarray analysis showed miR-24-1 were up regulated upon 5-Aza-CdR therapy in pancreatic cancer PANC-1 cells compared to control cells ( Lee et al., 2009).

**Table 4:** Top 50 miRNAs associated with 5-Aza-2′-deoxycytidine were predicted by GISMMA based on Dataset 1.

miRNA	Evidence	miRNA	Evidence
hsa-mir-21	26198104	hsa-mir-518c	unconfirmed
hsa-mir-324	unconfirmed	hsa-mir-200b	23626803
hsa-mir-23a	unconfirmed	hsa-let-7d	26802971
hsa-mir-24-1	26198104	hsa-mir-501	unconfirmed
hsa-mir-27b	26198104	hsa-mir-1226	unconfirmed
hsa-mir-27a	26198104	hsa-mir-200c	23626803
hsa-mir-638	26198104	hsa-mir-99b	unconfirmed
hsa-let-7a-1	unconfirmed	hsa-mir-181a-2	26198104
hsa-mir-124-3	23200812	hsa-let-7e	22053057
hsa-mir-124-2	23200812	hsa-mir-132	unconfirmed
hsa-mir-124-1	unconfirmed	hsa-mir-203a	26577858
hsa-let-7b	26708866	hsa-mir-409	unconfirmed
hsa-mir-18a	unconfirmed	hsa-mir-337	unconfirmed
hsa-mir-24-2	26198104	hsa-mir-1915	unconfirmed
hsa-mir-17	26198104	hsa-mir-128-2	unconfirmed
hsa-mir-181a-1	26198104	hsa-mir-128-1	unconfirmed
hsa-mir-663a	unconfirmed	hsa-mir-320a	26198104
hsa-let-7a-3	26227220	hsa-mir-181b-2	unconfirmed
hsa-let-7a-2	unconfirmed	hsa-mir-181b-1	unconfirmed
hsa-mir-126	26198104	hsa-mir-222	unconfirmed
hsa-mir-26a-1	unconfirmed	hsa-mir-26a-2	unconfirmed
hsa-mir-15b	unconfirmed	hsa-mir-328	unconfirmed
hsa-mir-16-1	26198104	hsa-mir-16-2	26198104
hsa-mir-125b-2	26198104	hsa-mir-29a	26198104
hsa-mir-125b-1	26198104	hsa-let-7c	unconfirmed


*The top 1–25 miRNAs are shown in the first column while the top 26–50 in the second. As a result, 7 and 25 out of top 10 and top 50 were confirmed by the known databases or experimental literatures, respectively.*

The whole prediction list of all candidate small molecule-miRNA pairs in Dataset 1 was provided in **Supplementary Table 1**, which was ranked in a descending order according to the association scores resulted from GISMMA. It is hoped that the ranked list can be useful in guiding biological experiments, and can be verified by more experimental results in the future.

## Discussion

This paper presented a graphlet interaction based method GISMMA to infer the potential associations between small molecules and miRNAs by combining small molecule similarity, miRNA similarity and known associations between small molecules and miRNAs. In GISMMA, we used a similarity network to represent the small molecules and used another similarity network to represent the miRNAs. An edge with a weight of the similarity value between two nodes was ploted when there was similarity between the two nodes, otherwise not. We utilized graphlet interaction to measure the complex relationship between two nodes in the network, where the graphlet is defined as a type of non-isomorphic subgraph ( Wang et al., 2014). Then, we counted each graphlet interaction isomer in a special pattern from the node having known associations to the node which does not have known associations. Therefore, we obtained a vector to describe the graphlet interaction between the two nodes. The correlation score between a small molecule and a miRNA can be computed through summing the weighted graphlet interaction isomers, where the weighs can be learnt from the known associations. The performance of GISMMA on predicting novel small molecule-miRNA associations was evaluated with four validation approaches that were global and two types of local LOOCV, as well as five-fold cross validation. The cross validation results were compared between GISMMA and SMiR-NBI, which showed the superior performance of GISMMA over SMiR-NBI. Besides, the ROC curves of SMiR-NBI are some unusual in **Figures 2, 3**, which may be attribute to that SMiR-NBI could not predict associated miRNAs (small molecules) for new small molecules (miRNAs). When ranking the test small molecule-miRNA pair with those candidate pairs for SMiR-NBI, we assigned fixed rank to those pairs that contain new small molecules (miRNAs) with an average number, which may cause the presence of line segments in the ROC curve. We have implemented cross validations on two datasets with different sizes. The results showed that GISMMA performed better on Dataset 1 than on Dataset 2, which could be resulted from two factors. The one is the more similarity information in Dataset 1. The other is that Dataset 1 contains those small molecules and miRNAs without any known associations, which often get lower association scores and lower rankings than the test sample. This could also make the AUCs higher. And we further executed case study for three small molecules using Dataset 1. The numbers of miRNAs that were validated to be related to these three small molecules by experimental literatures are in turn 30, 29 and 25 in top 50 miRNAs predicted by GISMMA. Via cross validations together with case study, we can see that GISMMA is well-performed and reliable in predicting new associations between small molecules and miRNAs. Furthermore, a list of all predicted small molecule-miRNA associations was provided, which would be favorable for the development of miRNA-targeted therapy and drug reposition. In detail, for a specific small molecule, we focused on the predicted miRNAs that are most possibly associated with this small molecule. These miRNAs might be related to some diseases that were not confirmed to be treated by this small molecule. Through regulating the expressions of these miRNAs, this small molecule could be used for the treatment of these diseases. Therefore, we believed that the prediction results of this work could offer some guidance for the experiment of drug reposition to some extent.

The outstanding performance of GISMMA can be attributed to several factors. Firstly, we mapped the similarity between small molecules and similarity between miRNAs into two networks, in which the similarity values were fully exploited to investigate the complex relationship between two nodes by measuring their graphlet interaction. Secondly, in GISMMA, not only direct but also indirect links were considered between the nodes in the counting of graphlet interaction isomers. Finally, the GISMMA is a bipartite method which combines miRNA network with small molecule network. It can be used to predict miRNAs associated with new small molecules without any known related miRNAs, as well as to predict small molecules associated with new miRNAs without any known related small molecules, because it computes the association score by combining the result calculated in the small molecule network with that in the miRNA network.

However, GISMMA still has some limitations. For example, the lack of the known association data, especially the presence of many new small molecules or new miRNAs that have no known associations, affected the performance to a large extent. It can be expected that the model will obtain better performance when more experimental datasets are produced in the future. Besides, the simple algorithm of averaging the scores from two networks to compute the final association score may cause bias to those pairs that can be predicted only in one network. Furthermore, GISMMA considered 4 nodes at most within a graphlet, which hindered it to contain more similarity information from more distant nodes. Finally, this model cannot be applied to the prediction of the association in which the small molecule and the miRNA are both new. We anticipate that more network-based methods could be developed to improve the prediction of novel small molecule-miRNA association. For example, Petri nets based models have been proved to be a useful tool for many prediction problems, inspired by the work in ( Russo et al., 2017), we could construct algorithm using Petri nets for the inference of potential small molecule-miRNA association.

## Author Contributions

N-NG implemented the experiments, analyzed the result, and wrote the paper. Y-ZS analyzed the result and wrote the paper. XC conceived the project, developed the prediction method, designed the experiments, analyzed the result, and revised the paper. ZM analyzed the result. J-QL analyzed the result and revised the paper. All authors read and approved the final manuscript.

## Conflict of Interest Statement

The authors declare that the research was conducted in the absence of any commercial or financial relationships that could be construed as a potential conflict of interest.
