# Supplementary material for: Prediction of Potential Small Molecule-Associated MicroRNAs Using Graphlet Interaction
Source: Front Pharmacol. 2018 Oct 15;9:1152. doi: 10.3389/fphar.2018.01152 (PMC6196296; doi:10.3389/fphar.2018.01152)
Supplement: Supplementary file 1 [file Table_1.DOCX]

**Prediction of potential small molecule-associated microRNAs using graphlet interaction**

**Na-Na Guan^1^, Ya-Zhou Sun^1^, Zhong Ming^1, 2^, Jian-Qiang Li^1,^ *, Xing Chen^3,^ ***

^1^College of Computer Science and Software Engineering, Shenzhen University, Shenzhen, 518060, China

^2^National Engineering Laboratory for Big Data System Computing Technology, Shenzhen University, Shenzhen, 518060, China

^3^School of Information and Control Engineering, China University of Mining and Technology, Xuzhou, 221116, China

*** Correspondence:**Xing Chen, Jian-Qiang Li

[xingchen@amss.ac.cn](mailto:xingchen@amss.ac.cn); [lijq@szu.edu.cn](mailto:lijq@szu.edu.cn)

**Supplementary Information**

**Supplementary Table 1.** We applied GISMMA to prioritize all the candidate small molecule-miRNA pairs by using all the known small molecule-miRNA associations as training samples. This prediction result is released for further experimental validation and research.
